# Recent Advancements in Plastic Packaging Recycling: A Mini-Review

**DOI:** 10.3390/ma14174782

**Published:** 2021-08-24

**Authors:** Valentina Beghetto, Roberto Sole, Chiara Buranello, Marco Al-Abkal, Manuela Facchin

**Affiliations:** 1 Department of Molecular Sciences and Nanosystems, University Ca’Foscari of Venice, Via Torino 155, 30172 Mestre, Italy; Roberto.sole@unive.it (R.S.); chiara.buranello@unive.it (C.B.); 870009@stud.unive.it (M.A.-A.); manuela.facchin@unive.it (M.F.); 2 Crossing S.r.l., Viale della Repubblica 193/b, 31100 Treviso, Italy

**Keywords:** waste valorization, plastic waste, circular economy, recovery, recycling

## Abstract

Today, the scientific community is facing crucial challenges in delivering a healthier world for future generations. Among these, the quest for circular and sustainable approaches for plastic recycling is one of the most demanding for several reasons. Indeed, the massive use of plastic materials over the last century has generated large amounts of long-lasting waste, which, for much time, has not been object of adequate recovery and disposal politics. Most of this waste is generated by packaging materials. Nevertheless, in the last decade, a new trend imposed by environmental concerns brought this topic under the magnifying glass, as testified by the increasing number of related publications. Several methods have been proposed for the recycling of polymeric plastic materials based on chemical or mechanical methods. A panorama of the most promising studies related to the recycling of polyethylene (PE), polypropylene (PP), polyethylene terephthalate (PET), and polystyrene (PS) is given within this review.

## 1. Introduction

In recent years, the health of our planet has become a problem of crucial importance, with plastic recovery and disposal being of primary relevance [1].

Since the introduction of Bakelite in 1907 by Leo H. Baekeland, the first fully synthetic polymer, the plastic industry has evolved to revolutionize the way we live [2,3,4,5].

**Figure 1:**
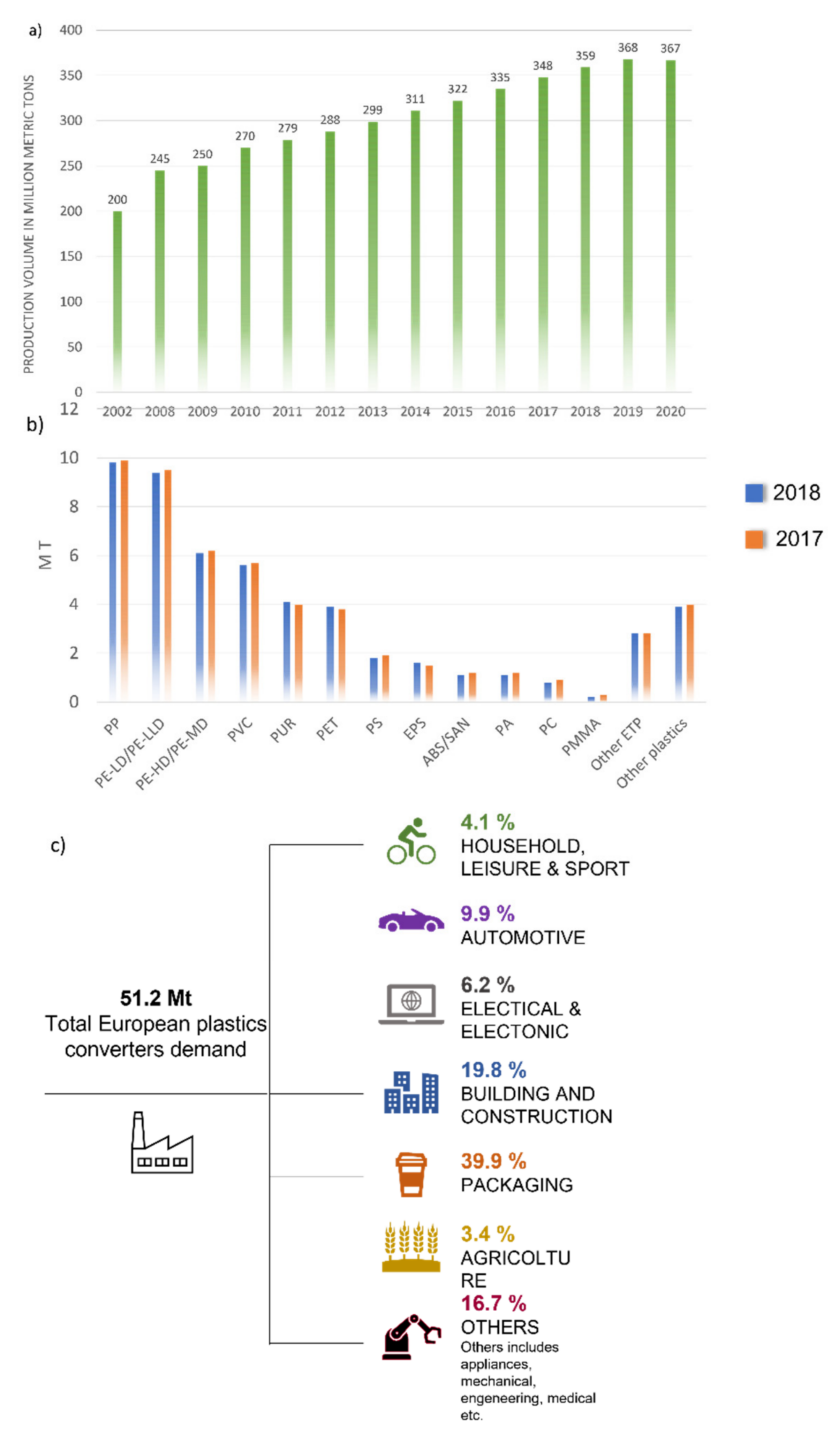
(**a**) World polymer production in metric tons; (**b**) distribution of main polymers produced; (**c**) 2018 European plastic converter demand and use.

Polymers and plastic products own their well-known ubiquity and massive use to their excellent chemical–physical properties, which guarantee light weight, low price, and endurance [6]. Thanks to their great versatility, plastics are among the most used materials and find applications in many industrial sectors such as packaging, automotive vehicles, construction, and electronic devices [1,7,8]. Worldwide, over 360 Mt of fossil-based polymers are produced yearly, with an annual growth rate of 8.4%, two times higher than world global gross growth rate of production over the same period [5] (Figure 1a). The European plastic converter demand in 2018 reached 51.2 Mt, mainly to produce polyethylene (PE), polypropylene (PP), polyvinylchloride (PVC), polyethylene terephthalate (PET), and polystyrene (PS) (Figure 1b). These are mainly employed for packaging (39.9%), construction (19.8%), automotive vehicles (9.9%), and electronic devices (6.2%) [9] (Figure 1c).

A gradual switch to biobased plastics has been witnessed by the increasing use at an industrial level of alternative raw materials [10,11] such as polylactic acid (PLA) [12], polybutyl succinate (PBS) [13,14], polyhydroxyalkanoate (PHA) [15,16,17], and polyethylene furanoate (PEF) [18,19,20], together with different composite materials produced from starch [21,22,23,24], CMC [25,26,27,28,29,30], wood [31,32], lignin [33,34], and many different agro-industrial wastes [35,36,37].

**Figure 2:**
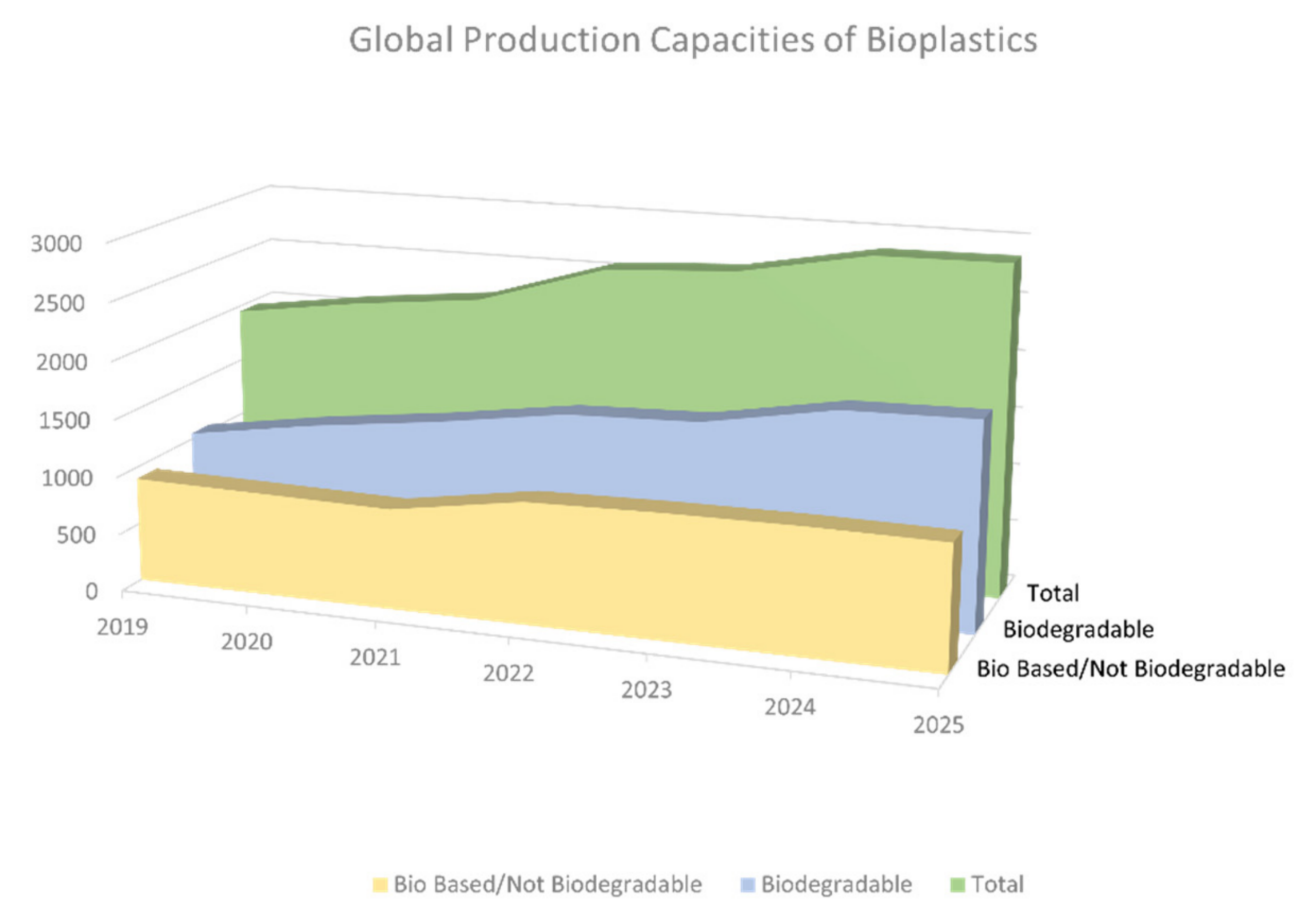
Projection of world global production capacity of bioplastics by 2024.

Nevertheless, 99% of plastics produced today are fossil-based polymers, and they will continue to play an important role in many manufacturing compartments for a long time. In fact, according to the 2020 European Bioplastics report, the EU total production capacity of biopolymers is expected to reach 2.45 Mt by 2024 (Figure 2), which is far lower than the plastic market needs [38].

The large gap between market demand and biobased plastics available today clearly shows the complexity of the problem and that all alternatives to approach the problem of plastic use and recycling must be pursued to reduce the environmental impact of polymers and plastic waste. In a recent article by Mendes and coworkers, the benefits of the use of bioplastics for the packaging industry were analyzed with the intent of delivering a guide for the design of more sustainable packaging to food packaging designers and producers [39]. The authors concluded that, from a climate point, the use of biobased plastics contributes to the generation of more sustainable food packaging compared to fossil-based ones; however, on the other hand, the relevance of some environmental problems originating from biobased plastics, such as eutrophication, use of water and pesticides, and effects on biodiversity, significantly reduces their environmental benefits.

**Figure 3:**
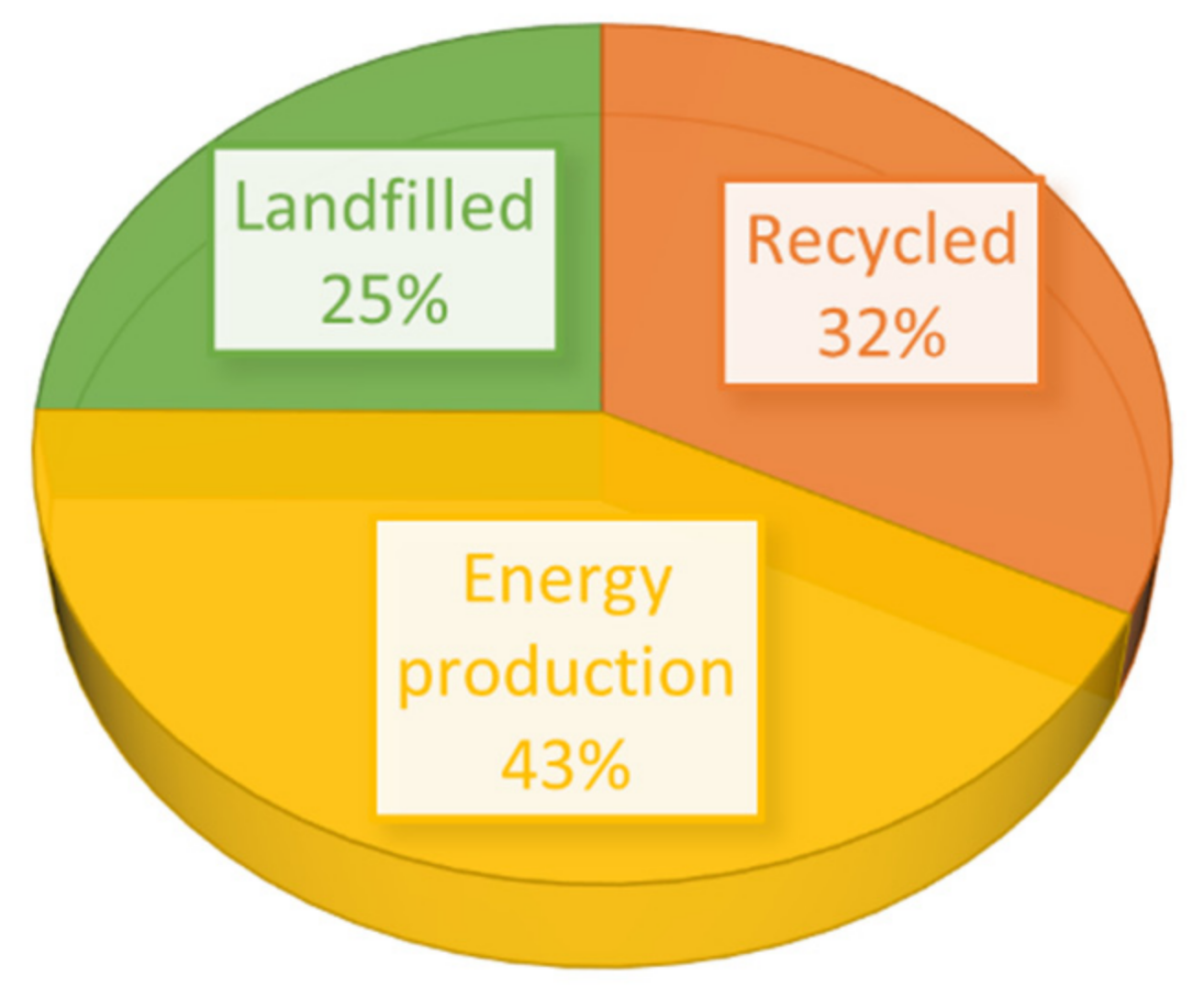
Reuse of recovered post-consumer plastic waste.

Additionally, fossil-based plastics are generally scantly biodegradable and accumulate in the environment, posing serious waste management problems. Over the last 65 years, approximately 8300 Mt of fossil-based polymers were produced, 4900 Mt of which were landfilled, incinerated, or dispersed in the environment [5,40]. Thus, oceans, animals, and humans are inevitably exposed to different sources of contamination from plastic waste [41,42,43,44,45,46]. Climate changes, environmental modifications, and health pandemics are becoming more and more frequent, showing that humanity will have to rethink its unsustainable growth [47,48] by adopting a circular economy approach to resource consumption through eco-design, recovery, and recycling of polymeric materials with an integrated approach [49,50,51,52,53]. Circular economy is pushing toward a radical change in production and waste management to reduce water, waste, and energy consumption and to achieve zero-waste manufacturing cycles [10,54,55,56,57]. In this frame, European countries have developed different waste management systems and recycling techniques [58,59,60,61,62,63,64]. Nevertheless, a great part of post-consumer managed plastic is currently sent to incineration or landfill, while mismanaged waste is either discarded into the environment or is inadequately disposed of, potentially ending up in the ocean [46]. From 2006 to 2018, the amount of recycled post-consumer plastic waste doubled, reaching 32.5% (29.1 Mt), while 42.6% was used for energy production and 24.9% was landfilled [9] (Figure 3).

In 2018, 5 Mt of plastic waste was recycled in Europe, 80% of which re-entered the EU as secondary materials, while the remaining 20% was exported outside the EU. The main industrial uses of recycled plastics in the EU are building and construction (46%), packaging (24%), agricultural applications (13%), and others (17%) [9].

Plastics may be subdivided into three categories: plastics in use, managed post-consumer plastic waste, and mismanaged plastic waste [65,66]. Managed plastic waste is generally disposed of by recycling, although a substantial gap exists between the quantity of plastic produced each year and the quantity of plastic thrown away since, depending on the type of product, there will be different storage and use times. Packaging products end their lifecycle generally in less than 1 year, while materials used for the construction and transport industry may last much longer. This means that the amount of waste produced each year is less than the amount of plastic in use. In 2015, 407 Mt of primary plastic entered the use phase, while only 307 Mt exited the use phase, with a consequent increase of 100 Mt of plastic in use [5].

According to the literature, it was estimated that, in 2010, between 4.8 and 12.7 Mt of plastics were leached into the ocean, predicting that, with inadequate waste management strategies, these numbers will increase by an order of magnitude by 2025 [46,67]. On this note, in January 2018, the European Commission issued the “European strategy for plastics in a circular economy” [68], including the ambitious target to make all plastics in EU recyclable by 2030. Soon after, in March 2018, China banned imports of plastic, generating a decrease in plastic waste export from EU of 39%, thereby overloading the EU waste management system and incinerators [65,69].

**Figure 4:**
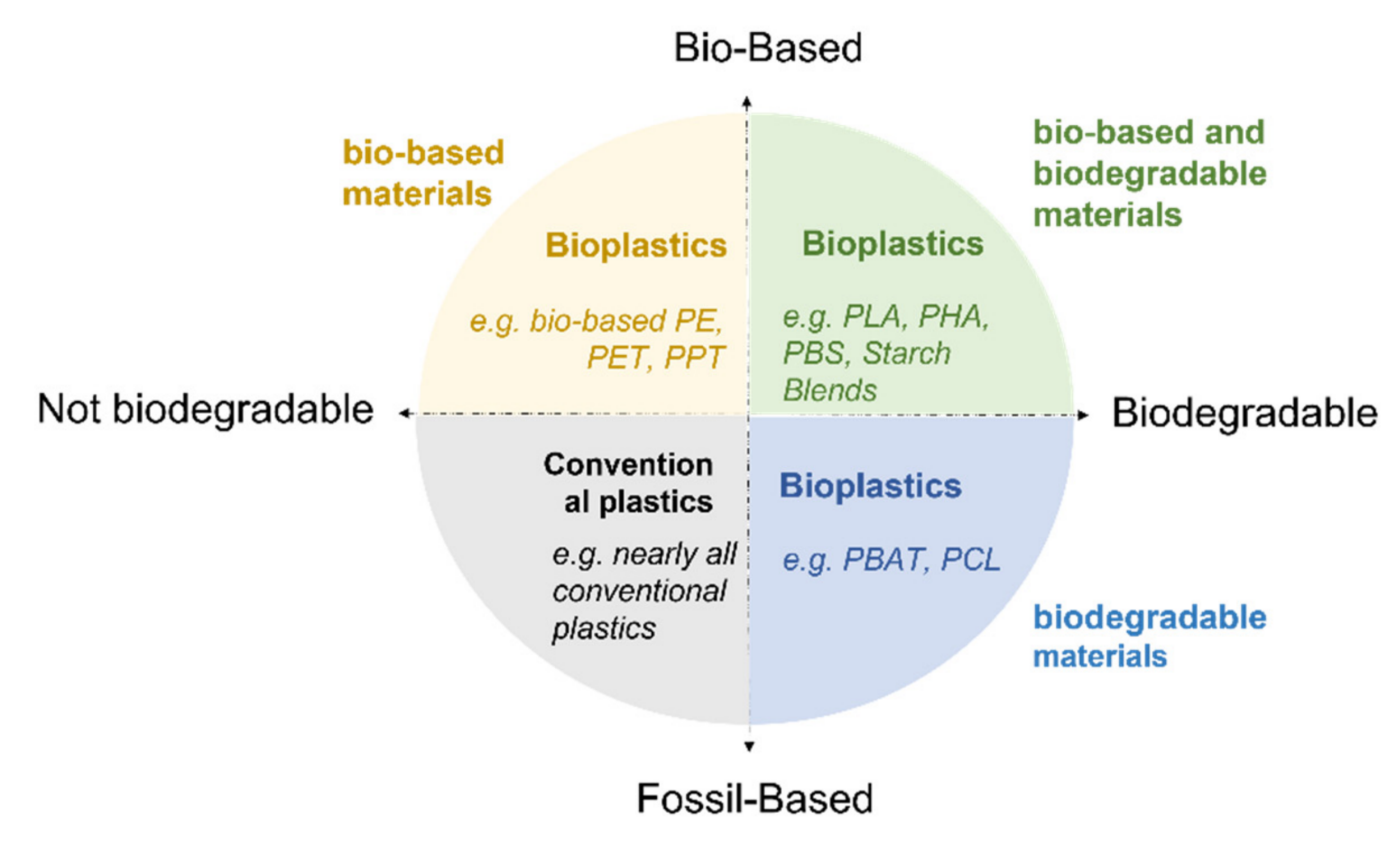
Examples of biobased and fossil-based polymers subdivided into biodegradable and not biodegradable.

To reduce the amount of plastic waste disposed in landfills or incinerated, there are two main strategies: the use of biodegradable biobased plastics (as mentioned above) [38,70] and recycling [71,72,73,74]. It should be reaffirmed that not all biobased polymers are biodegradable, while some fossil-based ones are, as clearly reported in Figure 4.

Moreover, the recovery and recycling of biobased polymers is a relatively new issue and is still the object of studies compared to fossil-based polymers [39,75]; thus, different strategies will need to be put in place to implement the environmental sustainability of polymer manufacturing and recycling. According to the recent Circular Economy Package EU legislation and a paper by Briassoulis and coworkers, mechanical recycling is the best alternative for the valorization of both post-consumer fossil-based and biobased polymer waste, followed by chemical recycling [75,76].

The topic of sustainable manufacturing of plastics and packaging is so important that, from a research on Google Scholar using as key words “sustainable plastics”, “recycled plastic”, and “plastic recycling techniques”, a total of almost 95,000 papers were published between 2019 and 2021. This mini-review intends to give an outlook on different mechanical and chemical recycling techniques, giving a general panorama of the state of the art and recent innovative solutions by focusing mainly on papers published in the last 12 months relevant to plastic packaging. The scope of the work is to give a general overview of most recent technologies for the recycling of post-consumer packaging waste (PP, LDPE, HDPE, PET, and PS) to be used as secondary materials for the manufacturing of different materials. Since it is possible that the EU will implement plastic recycling up to 100% by 2050, avoiding the use of virgin naphtha for its production, the use of plastic waste as a source of energy seems bound to assume a minor importance in the future, while recycling of polymers to produce high-value products will be of strategic importance. For this reason, techniques to produce energy from plastic waste will not be discussed in this mini-review. The authors believe that a good understanding of the possible alternatives to plastic recycling and valorization, together with the difficulties encountered in sorting and reprocessing of post-consumer plastic waste, should help the industry, as well as end users, to adopt more responsible behavior and, consequently, promote the introduction of environmentally sustainable solutions.

## 2. Overview of Plastic Recycling Techniques

**Scheme 1:**
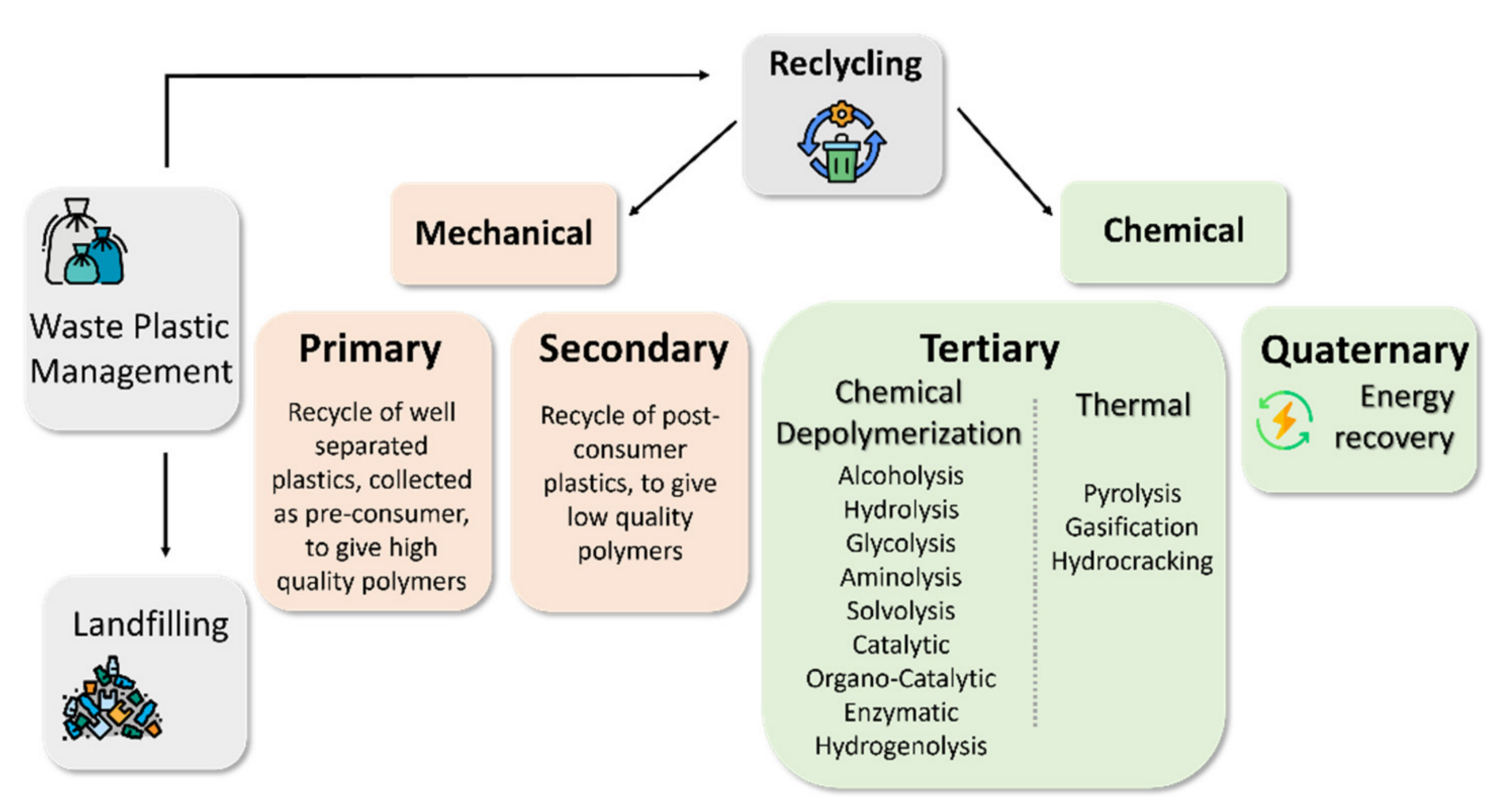
Overview of plastic recycling techniques.

The word recycling refers to a set of modifications and transformations (mechanical treatment, chemical treatment, or heating) required to recover feedstock from a previously processed polymer which can be reused by the industry [73,77,78]. Plastic recycling methods available today are classified in primary to quaternary processes [79,80] (Scheme 1).

Specifically, primary processes allow recovering and recycling pre-consumer or pure polymers which can be reused for the same scope. Secondary processes start from recovered post-consumer polymeric waste, which is sorted, trimmed, and re-extruded, giving a product with reduced physical–mechanical characteristics compared to the starting polymer, which in most cases cannot be reused for the same scope. Primary and secondary recycling represents physical processes that can be repeated several times. Tertiary processes adopt chemical recycling starting from polymers which may no longer undergo mechanical recycling, while quaternary ones are used for energy production. Polymers and plastics sent to landfill (end-of-life plastics) lose their value and become waste.

Different techniques adopted for plastic waste separation, processing, and possible reuse as secondary materials depend on the type of waste recovered. A first important distinction should be made between thermoplastic and thermoset polymers. Thermoplastics are usually processed by extrusion, as these polymers melt when heated and harden when cooled. A great advantage of thermoplastics is that the extrusion process can be repeated many times. The most used thermoplastics are PP, PET, LDPE, HDPE, PVC, and PS. Adversely, thermosets may not be reprocessed by extrusion since, when heated, an irreversible chemical reaction takes place. Main thermoset plastics are polyurethanes (PUR), resins (epoxy, phenol-formaldehyde, and polyester), and vulcanized rubber, widely used by the automotive and electronic industry. The most abundant polymers in post-consumer waste are polyolefins (PP, LDPE, HDPE, PET, and PS) used for packaging [58,81,82], with a consumption of over 23 Mt only in the EU in 2020.

## 3. Primary and Secondary Recycling

**Scheme 2:**
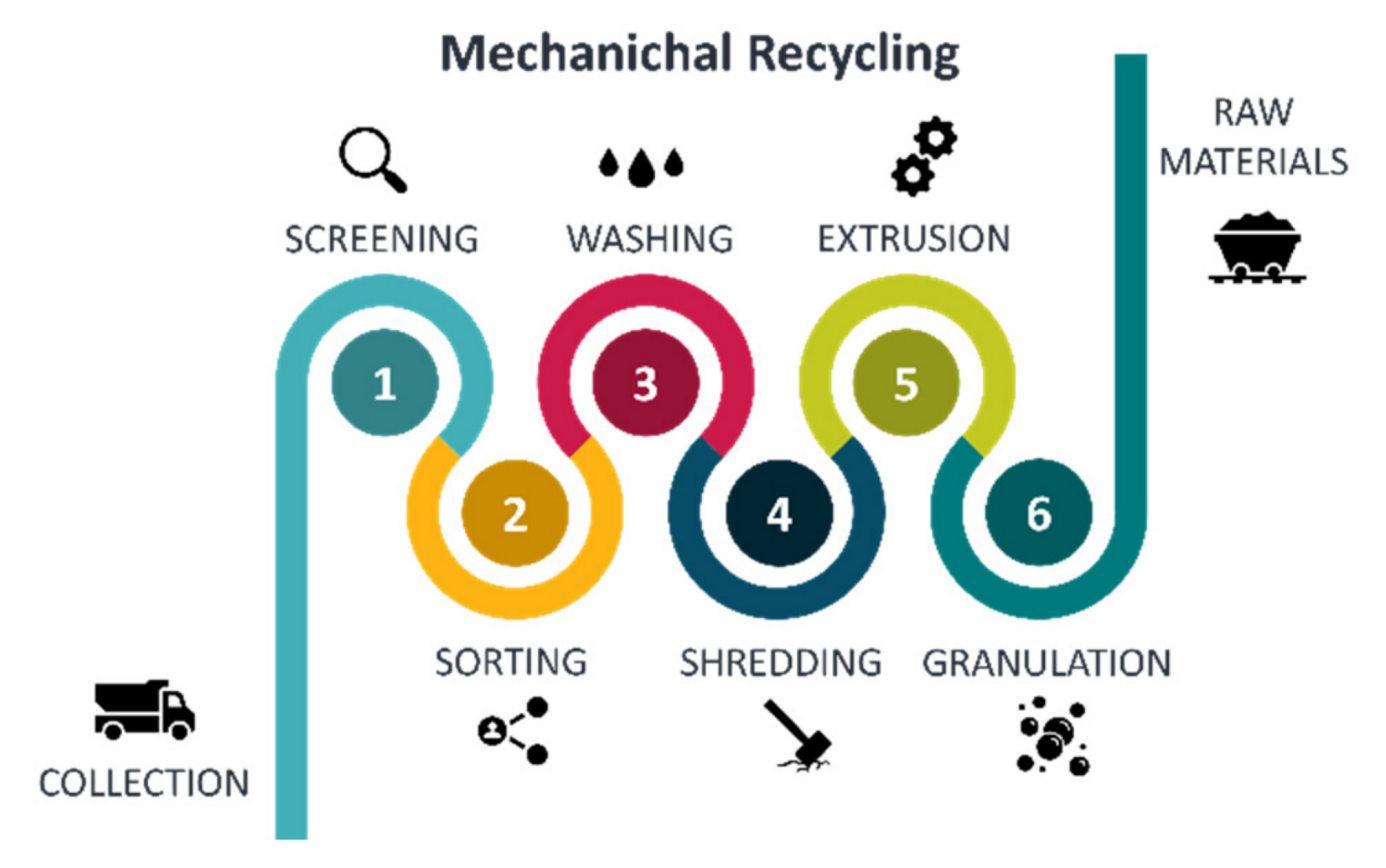
General scheme of primary and secondary recycling processes.

Mechanical recycling is the main and most widely used technology for plastic recycling, consisting of several steps, including collection, screening, automatic or manual sorting, washing, shredding, extrusion, and granulation [83,84,85,86] (Scheme 2). Mechanical recycling is classified as primary or secondary according to the type of starting material being processed. Primary recycling gives the highest-quality recycled polymers and starts from closed-loop recycled products such as PET bottles or byproducts collected by manufacturing industries as pre-consumer well-separated material.

Secondary processes instead recover post-consumer plastics and, therefore, generate lower-quality polymers. It must nevertheless be considered that, from an economic standpoint, these processes have a reduced complexity and overall limited costs, generating significant income and reduced CO_2_ production. According to the Ellen MacArthur foundation report, plastic production and incineration of plastic waste are estimated to produce over 400 Mt of CO_2_ yearly [87,88]. Thus, recycled plastics can reduce fossil-fuel consumption and CO_2_ emissions. According to estimates by Rahimi and coworkers [89], the adoption of plastic waste recycling worldwide would allow saving about 3.5 million barrels of oil each year.

Mechanical recycling generally includes four main steps: (i) screening and sorting; (ii) shredding; (iii) washing and drying; (iv) melting and reprocessing (Scheme 2).

Screening and sorting of plastic waste is a fundamental step for the recyclability of the different plastics and the quality of the final polymer. This step is challenging, considering that the separation of mixed plastic waste often involves the combined use of different technologies [90,91].

To achieve an adequate separation of a specific polymer within a flow stream containing many different components (plastics, as well as metals, paper, organic residues, and dirt), characteristics of the final product must be accurately considered such as purity and destination. This will allow defining the best separation strategy to achieve high selection. Important properties commonly employed for plastic separation are magnetic or electric properties, particle size, density, and color. Relying on these properties, many different separation techniques have been developed such as dry or wet gravity separation, electronic or magnetic density separation, flotation, and sensor-based sorting together with auxiliary segregation techniques such as magnetic or eddy-current separation. These segregation methods are briefly described, mainly focusing on recently implemented technologies for PE, PP, PET, and PS recovery.

**Figure 5:**
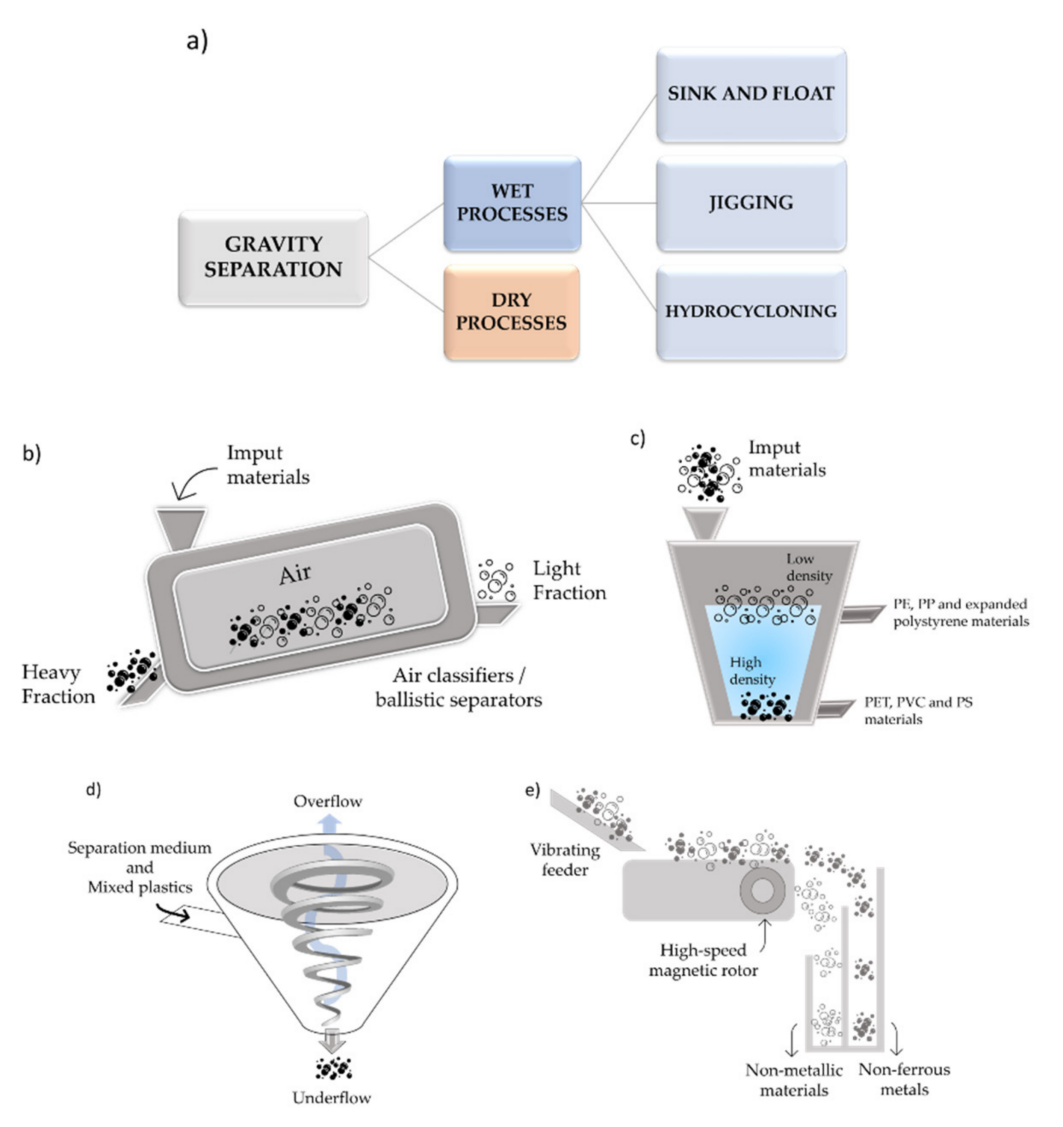
(**a**) Different methodologies of gravity separation; (**b**) dry segregation; (**c**) sink and float separation; (**d**) hydrocycloning; (**e**) eddy current separator.

Gravity separation is a consolidated methodology that may be carried out in a dry environment (dry process) or in the presence of water (wet process) [63,92] (Figure 5a). Dry segregation techniques employ air classifiers or ballistic separators in which air is used as the medium to separate lighter materials from heavier ones. They can be positioned at the beginning of the process or at the end, to segregate end-of-life plastics from main plastic streams (Figure 5b). Wet gravity separation includes sink and float, jigging, and hydrocyclone techniques.

With sink and float separation, polymers are separated into two different streams depending on whether they have a higher or lower density than water. Materials such as PET, PVC, and PS will sink, while others such as PE, PP, and expanded polystyrene will float (Figure 5c). This type of separation guarantees an effective first separation, but it is not adequate to produce high-quality secondary materials and needs to be combined with other separation techniques [93,94,95].

Zhang and coworkers developed a pretreatment of PET via preliminary NaOH and ethanol hydrolysis to promote plastic flotation. Optimal conditions allowed the quantitative recovery of highly pure PET fractions [96].

Heidarpour and coworkers reported the influence of microwave irradiation in the presence of chemical additives such as PEG-400, methylcellulose, or tannic acid on the float–sink behavior of polyoxymethylene, polycarbonate, and polyvinyl alcohol. According to this study, microwave irradiation reduced the contact angle values of tested plastic surface in the presence of chemical additives (depressant) by implementing their sink–float separation capacity, thereby increasing their hydrophilicity [97]. The authors mention the possibility of using this technology for whichever plastic material.

Jigging is one of the oldest gravity separation techniques and is similar to dry gravity methods where, in most cases, water is used instead of air [90,94]. A water stream is pushed up and down by pistons, and plastics are separated mainly depending on their morphological and physical characteristics.

Hydrocycloning is based on centrifugal and centripetal forces together with the fluid resistance of different materials processed (Figure 5d). New trends in hydrocycloning separation focus especially on the recovery of precious metals from electronic device waste [98,99], and it seems to be a very valuable tool for more sustainable separation of plastic waste from metals.

The eddy current separator is made of a high-speed magnetic rotor which generates an electric current, the so-called eddy current, used to remove nonferrous metals (aluminum and copper) from waste plastic, glass, and paper, among others (Figure 5e) [100]. These separators are generally located at the beginning of the recycling process.

With a separator and drum screen, plastics are fed into a large rotating drum where materials are separated by size, thanks to holes in the drum, so that only smaller particles pass through and are separated from larger ones.

Different gravity segregation methods were analyzed by Nie and coworkers for the sustainable recovery and recycling of high-value metals from waste printed circuit board (WPCBs) [101]. This study analyzed the dynamics and statics of gravity concentration methods. The settling velocity of three kind of particles was studied, demonstrating that the stratification by density is spontaneous and can achieve the lowest potential energy. The concentration of differently sized metal particles could be effectively enriched, and the metal purity increased from 56.5% to 68.2% for decreasing particle size, albeit with a modest decrease in yield (from 86.41% to 83.04%). No recent papers were found for the use of innovative solutions for the recovery of PE, PP, PET, or PS by jigging, hydrocycloning, eddy current separation, and drum and different gravity segregation techniques, but they were reported to give a general overview of different separation technologies available.

Optical sensors are used for the characterization of plastic stream in a continuous manner where air jets allow for separation. Optical sensors may be subdivided in molecular spectroscopies and atomic spectroscopies [102], the prevalently used Raman spectroscopy (RS) [103], Fourier-transform infrared spectroscopy (FTIR) [96], near-infrared spectroscopy (NIRS) [104], and terahertz spectroscopy (THz) [105], and elemental spectroscopies such as laser-induced breakdown spectroscopy (LIBS) [106] and X-ray fluorescence spectroscopy (XRFS) [102].

Bobulski and coworkers implemented new portable devices for computer image recognition in combination with artificial intelligence for waste recognition and easy municipal waste separation. The devices were used both at home and in waste sorting plants, and they could be a very useful tool for an efficient and economically sustainable separation of plastic waste stream [107].

Most companies use a combination of different separation techniques to obtain sufficiently pure polymers from post-consumer plastic waste. The purity of the finished product depends on an adequate compromise between costs and benefits, and this leads to purities ≤95% which require further separation and purification steps. Sorting technologies reported above are generally inadequate for the separation of complex materials such as multilayered packaging or fiber-reinforced composites; therefore, these materials are generally incinerated for energy recovery or landfilled as end-of-life plastics.

Innovative recycling methods such as selective polymer dissolution were demonstrated to be efficient in extracting different polymers and fibers from multilayered films and composite materials [108]. In fact, Knappich and coworkers reported the efficient recovery and recyclability of epoxy and polyurethane resins from carbon fiber-reinforced plastics with different proprietary CreaSolv^®^ formulations at a laboratory scale.

Multi-material plastic waste separation technologies are also being developed to enable a proper sorting of composites, which will generate new value streams to recover and recycle plastics which are today incinerated or landfilled [109]. Many approaches have been tested, for example, for the separation of polyester from cotton fibers to recycle textile waste. Solvent-based technologies are an interesting solution, with the possibility of selecting specific solvents which may solubilize either cotton or polyesters [110]. A crucial aspect for industrial success and applicability is the nature of the solvent in terms of volatility, flammability, toxicity, and recyclability [111].

Once the mechanical separation is complete, the materials are shredded by passing them through a system of rotating blades. The obtained flakes are then sorted by size with a grid, washed and dried, made ready for reprocessing by extrusion or agglomeration, and sold.

Agglomeration is generally used to reprocess plastic films which are cut in small pieces, heated by friction and water-cooled. The agglomerates are usually combined into plastic flakes and pelletized by extrusion. Agglomeration is highly energy-consuming and, therefore, less widespread [90].

Extrusion remains the most widely used method for processing both virgin and recycled plastic. Plastic flakes are fed into the extruder and pushed by a screw into a heated cylinder, thus melting the plastic. At the end of the extruder, a pelletizer cools and cuts the final polymer into pellets.

Both shredding and extrusion may lead to partial degradation of the polymer due to chain scission and thermo-oxidative reactions, reducing the polymer chain length and, consequently, its mechanical properties [112,113]. Moreover, impurities deriving from other packaging components further contribute to the diminished physical–mechanical characteristics of reprocessed plastics [104].

A detailed study was published by Eriksen and coworkers on the thermal degradation, processability, and mechanical properties of re-extruded PET, PE, and PP from post-consumer waste. PET is well suited for closed-loop recycling to meet bottle and food-grade PET quality, although moisture control is a key requirement when reprocessing PET into products. For this polymer, degradation, which generally occurs during recycling by extrusion, may be avoided by careful decontamination. The quality of reprocessed PE samples from non-food bottles strongly depends on the presence of impurities from other polymers and from lids and labels. PE reprocessing by extrusion suggested that closed-loop recycling may be achieved with selected PE bags with low levels of polymer cross-contamination. Adversely, PP reprocessed by extrusion showed low mechanical properties with large variations in impact strength, reducing possible applications of reprocessed PP. Thus, the heterogeneity of PP waste, even if food packaging is managed separately, as well as polymer degradation during recycling, represents crucial limitations for PP waste recycling [114].

A possible remedy to downgrading due to extrusion was reported for the first time by Wang and coworkers. The authors reported a process to modify polyolefins from post-consumer plastic waste via a one-step radical grafting and cross-linking process, producing covalent adaptable networks or CANs [112]. This procedure relies on the functionalization of polyolefins with polar reagents, which modify the properties of the starting material, thus imparting new characteristics such as wettability, printability, and compatibility with other polymers. Upcycling of LDPE from plastic bags was achieved by free-radical reaction in a twin-screw extruder in the presence of maleic anhydride and butanediol. PE-CANs showed higher solvent resistance, tensile strength, and modulus compared to virgin PE due to the presence of cross-linking bonds generated during the extrusion process. Upcycling of post-consumer plastic waste by reactive extrusion is an interesting area of research which will surely receive much attention in the future; however, characteristics of CAN polymers must be acquired to define new possible manufacturing applications [115].

## 4. Chemical Depolymerization

In addition to mechanical methods, recycling can be performed via chemical depolymerization [111,116].

Chemical recycling has great potential in the circular economy of plastics; it can close the loop by producing starting monomers from the polymers that may be reprocessed to produce high-value-added chemicals [70]. It is estimated that, by 2050, almost 60% of plastic production can be based on recycled products [117]. Millions of euros are being invested to enhance chemical recycling and other cutting-edge technological solutions with the aim of producing 1.2 Mt of recycled plastic in EU by 2025 and 3.4 Mt by 2030 [9].

Chemical recycling methods are classified according to reaction conditions into solvolysis (hydrolysis, methanolysis, and glycolysis), catalytic depolymerization, and enzymatic depolymerization [83,84,118,119,120,121,122,123,124,125,126,127]. Only main innovative solutions devised in the last year for plastic packaging chemical recycling are analyzed below i.e., PE, PP, PET, and PS.

### 4.1. Solvolysis

Solvolysis involves the breaking of the hydrolyzable bonds of a polymer in the presence of an alcohol or water. It is rather frequent that, to improve reaction conditions, product selectivity, and yield, catalysts are used to promote solvolysis reactions [83,84,119,128].

#### 4.1.1. Hydrolysis

Hydrolysis reactions perform better from an environmental point of view but require higher energy consumption compared to other solvolysis methods [129]. They may be carried out in neutral, acidic, or alkaline conditions.

**Scheme 3:**
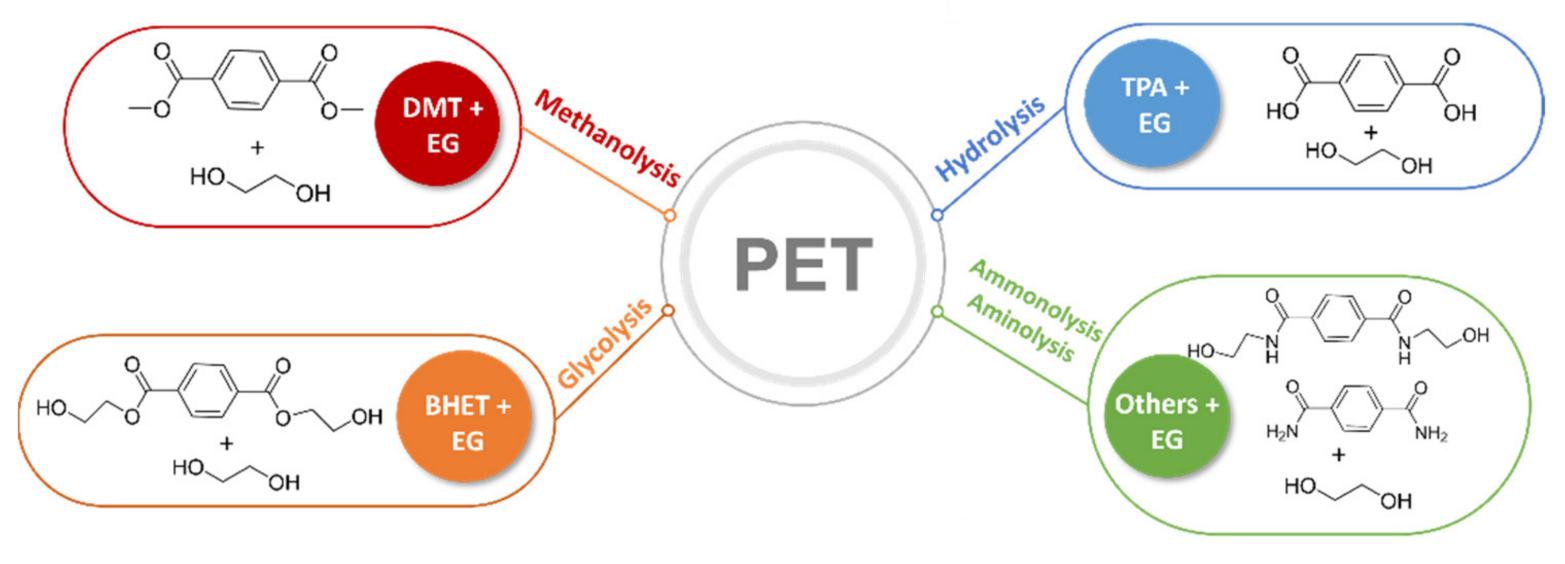
PET chemical recycling routes and product desired.

Neutral hydrolysis of PET has long been known and is generally processed in the molten phase, at temperatures above 245 °C with a water/PET (*w*/*w*) ratio above 5.1/1. A further improvement in the rate of the reaction may be achieved via the addition of catalytic amounts of alkali metal acetates, organophosphorus compounds, or zeolites [128]. Recently, Colnik and coworkers reported hydrolytic recycling of colorless and colored PET bottles in sub- and supercritical water with temperatures between 250 and 400 °C, in 1 to 30 min. Highest yields in terephthalic acid (TPA) were achieved at 300 °C in 30 min with purities near to 100% [130] (Scheme 3).

Interestingly, according to the work by Stanica-Ezeanu and coworkers, sea salt is an efficient neutral catalyst promoting PET degradation; by means of a mathematical model, it was estimated that, in tropical regions, only 72 years are necessary for spontaneous complete degradation of PET to occur [131].

Acid hydrolysis of PET proceeds by polymer dissolution in concentrated acids (H_2_SO_4_, H_3_PO_4_, and HNO_3_) and heating, leading to chain fragmentation at high temperature.

These processes have not been, to the best of our knowledge, the object of recent studies, probably due to their low environmental sustainability; therefore, they are not further discussed in this review.

Alkali-promoted glycolysis of PET has been widely reported using both inorganic and organic bases [132]. Due to the high quantities of alkali required and consequent environmental impact of the process, in this case, no innovative solutions were found in recent publications.

#### 4.1.2. Methanolysis

Methanol is widely used and is effective for the solvolysis of various polymers such as PET, polyamides, and polycarbonates. The majority of post-consumer recovered PET is currently reprocessed by mechanical recycling; however, this process leads to molar mass reduction and a consequent reduction in the physical–mechanical properties of the polymer, which is generally used to produce carpets (72%) [70], along with a small percentage of PET for bottle production [129]. Moreover, the commercial appeal of mechanical recycled PET depends on the price of oil; thus, when oil is available at prices below $65 per barrel, mechanically recycled PET is no longer competitive [70]. Chemical depolymerization to produce high-quality monomers and oligomers may be a solution to this problem.

The primary scope of PET chemical recycling is to regenerate TPA, dimethyl terephthalate (DMT), bis(2-hydroxyethyl) terephthalate (BHET), and ethylene glycol (EG) [133] or other chemical substances [134,135] (Scheme 3).

**Scheme 4:**
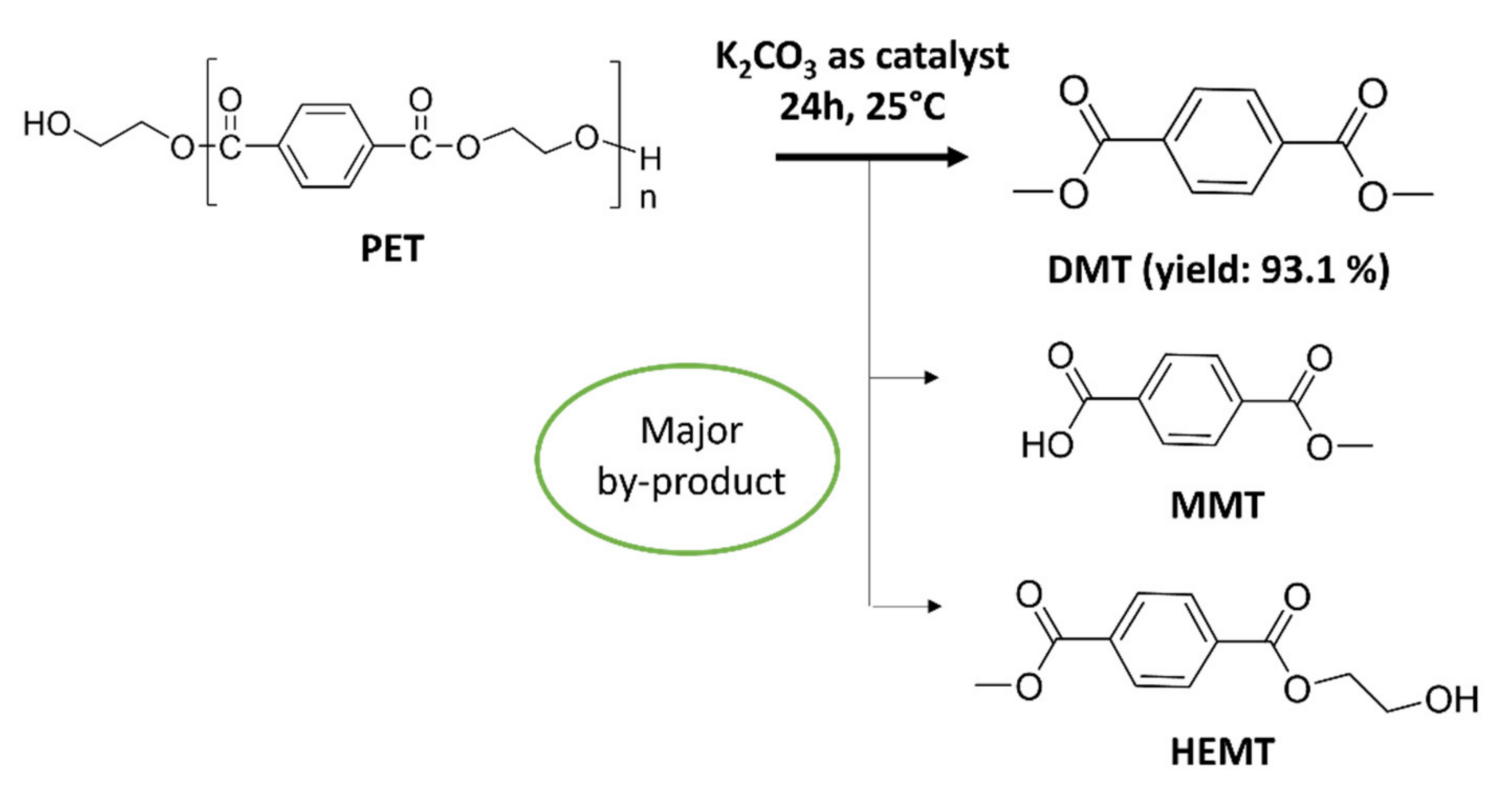
Low-energy catalyzed methanolysis of PET.

Methanolysis of PET is generally a degradation process performed at high temperatures (180–280 °C) and pressures (2–4 MPa), and the major products are DMT and EG [70,129], with high capital and operating costs. Recently, Pham and coworkers [124] developed a low-energy catalyzed methanolysis to convert PET into DMT at room temperature in the presence of K_2_CO_3_ as a catalyst. Despite the overall reaction time of 24 h, PET resins were completely decomposed into monomers with high selectivity in DMT with 93.1% yield at 25 °C. 2-Hydroxyethyl methyl terephthalate (HEMT) and monomethyl terephthalate (MMT) were the major byproducts collected after the reaction (Scheme 4).

Myren and coworkers described a new method for methanolysis of post-consumer PET waste in the presence of NaOH carried out in a microwave or electrochemical reactors. Under mild reaction conditions (85 °C, 40 min) overall yields in TPA of 65% were achieved under microwave irradiation [136].

Barnard and coworkers published a review in 2021 evaluating advantages and disadvantages of chemical recycling of PET based on the energy economy coefficient and environmental energy impact. Different technologies evaluated comprised neutral, acidic, or alkaline hydrolysis, enzymatic hydrolysis, solvolysis, glycolysis, and aminolysis. From the comparison of data collected, alcoholysis was the most energetically expensive process; moreover, the low boiling point of alcohols generally requires high-pressure reactors. On the contrary, methanolysis carried out in the presence of a nanodispersion of ZnO was found to be the least energetically expensive process for PET degradation, giving high-quality DMT [129,137].

Additionally, Zhang and coworkers proposed a novel, simple and economic hydrophilic modification of PET by surface alcoholysis in the presence of ethanol and a sodium hydroxide water solution, which influenced the wettability of PET and promoted sink–float separation from hydrophobic PS, PVC, and PMMA [96].

**Figure 6:**
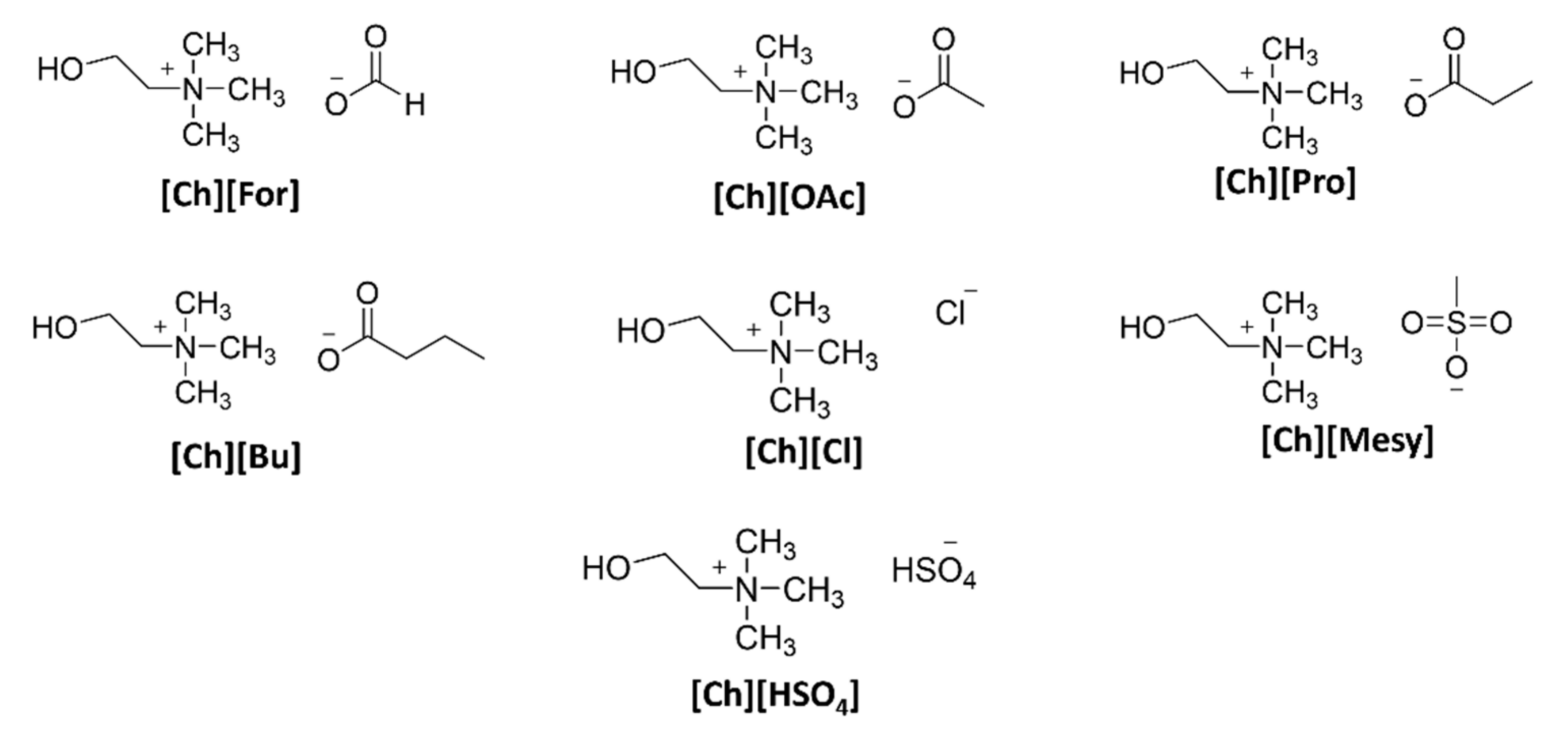
Main ILs reported in literature.

Another very interesting example of the methanolysis of PET was achieved in the presence of an organocatalyst prepared from very simple reagents such as tetramethyl ammonium hydroxide and dimethyl carbonate, [NMe_4_]^+^[OCO_2_Me]^−^, achieving good yields of DMT (≤75%) in mild reaction conditions (100 °C and 4 wt.% organocatalyst) [138]. Nevertheless, long reaction times (16 h), solvents, and product purification were necessary. Alternatively, imidazolium metal-based ionic liquids (ILs) can achieve a comparable or even better performance than [NMe_4_]^+^[OCO_2_Me]^−^ [139]. Main ILs reported in the literature are depicted in Figure 6.

#### 4.1.3. Glycolysis

Glycolysis was also verified to be a promising alternative with moderate energy and environmental impact [129]. Glycolysis produces the BHET monomer, which is a good starting material for PET upcycling. As reported by Lalhmangaihzuala and coworkers, glycolysis of post-consumer PET waste may be efficiently promoted by heterogenous catalysts prepared from orange peel ash. Total depolymerization of PET was detected within 90 min, producing BHET in 79% yield. The catalysts were recovered up to five times without significant deactivation. This study opens the way to a highly environmentally sustainable approach to post-consumer plastic waste recycling [127].

Organocatalyst-assisted glycolysis is considered a new frontier for a green approach to plastic recycling in comparison to conventional organometallic complexes [138,140]. Wang and coworkers [141] reported a very promising study on the glycolysis of PET using 1,3-dimethylimidazolium-2-carboxylate as an organocatalyst, achieving complete depolymerization in less than 1 h at 180 °C, with up to 60% yield in BHET recovered by precipitation from the reaction mixture upon cooling.

Alternatively, Fuentes and coworkers reported the glycolysis of PET bottles to BHET in the presence of catalytic amounts of different metal oxides (ZnO, CoO) obtained for the recycling of spent alkaline and lithium-ion batteries. Reactions were carried out in EG at approximately 200 °C for 2 h; in the best conditions, yields of the BHET reached 80% [126].

Functionalization of silica-coated, magnetic Fe_3_O_4_ nanoparticles, with an iron-containing ionic liquid, was recently employed for the glycolysis of PET to BHET. The advantage of these catalysts is in their high recyclability and ease of recovery due to their magnetic properties, and no traces of metals were found in the final products [142].

#### 4.1.4. Aminolysis

**Scheme 5:**
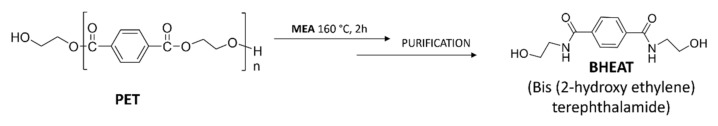
PET aminolysis via monoethanolamine (MEA).

While aminolysis presents the best energy and environmental parameters, the use of ammonium-based ionic liquids makes the production process more expensive [129,143,144]. The high temperatures involved in aminolysis are compensated for by very low depolymerization times due to increased reaction speed. Adversely, depolymerization by aminolysis of PET produces terephthalamides which have limited industrial applications. Different amines such as monoethanolamine (MEA) have been used for the aminolysis of PET with and without catalysts such as metal salts, quaternary ammonium compounds, and ionic liquids [145] (Scheme 5).

Catalyst-free, microwave-assisted aminolysis of PET proved to be an efficient method for the recovery of different terephthalamides starting from allylamine, ethanolamine, furfurylamine, or hexylamine with high selectively and yields. Terephthalamides were employed to produce good quality films [123]. Furthermore, aminolytic upcycling of PET post-consumer waste was achieved in the presence of different amino-alcohols in the presence of various organocatalysts to give diol terephthalamides, which were employed to produce poly(ester-amides) [146].

### 4.2. Catalytic Depolymerization

Plastic depolymerization may be carried out in the presence of different catalysts such as strong mineral acids, bases, organocatalysts, enzymes, and metal catalysts in homogeneous or heterogeneous phase [147].

#### 4.2.1. Enzymatic Catalysis

To date, the enzymatic activity of various microbial and fungal species has been tested for the degradation of various polymers [148,149]. As with chemical degradation, the major difficulty in the enzyme degradation of polymers such as PE and PP derives from their high hydrophobicity, stability, and inertness, and their reactivity may be implemented by UV or thermal oxidation pretreatments [150]. While PE and PP enzymatic degradation is still a very challenging topic, numerous hydrolytic enzymes have been identified and are efficient for PET degradation [151]. PET hydrolases represent one of the most recent breakthroughs in the depolymerization of post-consumer PET, allowing the recovery of terephthalic acid and ethylene glycol at industrial relevant scale [120]. Interestingly, Sadler and coworkers developed an innovative enzyme-catalyzed post-consumer PET hydrolysis with engineered *Escherichia coli* to produce vanillin [134].

These new technologies once more highlight the importance of the development of specifically devised new microorganisms and enzymes for plastic depolymerization. In this connection, Santacruz Juarez and coworkers reported the use of molecular docking simulation to predict affinity, strength, and binding energy between two molecules to analyze the activity of laccase (Lac), manganese peroxidase (MnP), lignin peroxidase (LiP), and unspecific peroxygenase (UnP), thereby helping in the development of new enzymes [152]. Data achieved showed that synergic enzymatic combination, as it normally happens in nature, boosts the catalytic efficiency by promoting sequential degradation processes. The use of microorganisms and enzymes has been widely studied with the intent to find an environmentally sustainable solution to microplastic and nanoplastic contamination. Taghavi reviewed the state of the art of plastic packaging biodegradation by living microorganisms reporting mechanisms of action, advantages, limitations, and technology readiness levels (TRL). The focus of this very important research area is a reduction in plastic pollution in the environment more so than its recovery and reuse; thus, it is not further analyzed in this paper [148].

#### 4.2.2. Hydrogenolysis

Hydrogenolysis is widely employed for the depolymerization of PET in the presence of hydrogen and homogeneous Milstein-type Ru–PNN complexes which are highly reactive toward the C=O double bonds of PET to give 4-benzenedimethanol (BDM) in 99% yield at 160 °C in 48 h (Table 1, entry 1), while they are ineffective in the presence of PP and PE [147,153,154,155]. More complex phosphine ligands have also been tested, but the economic viability on an industrial scale seems to be rather limited [147] (Table 1, entries 2–3).

Two very important studies have been published on the efficient conversion of post-consumer PET to benzene, toluene, and xylenes by reportedly “unlocking hidden hydrogen in the ethylene glycol part” with Ru/Nb_2_O_5_ catalyst [156,157]. The hydrogen is formed in situ during the reaction from ethylene glycol, and it appears that, in the presence of Ru/Nb_2_O_5_, two different pathways (decarboxylation and hydrogenolysis) compete to determine the selectivity toward alkyl-aromatic compounds (Table 1, entries 4–5) [156].

Solventless hydrolysis of PET bottles to TPA and ethylene has been selectively achieved by a carbon-supported single-site molybdenum-dioxo catalyst under 260 °C and 1 atmosphere of H_2_ with 87% yield. The catalyst exhibits high stability and can be recycled many times without loss of activity [158].

Hydrogenolysis of PET to liquid alkanes has been carried out under mild reaction conditions using ruthenium nanoparticles supported on carbon (Ru/C). Under optimal reaction conditions (200 °C, 20 bar H_2_, 16 h), PE was converted into liquid *n*-alkanes with 45% yield [159]. Another SnPt/γ-Al_2_O_3_ and Re_2_O_7_/γ-Al_2_O_3_ heterogeneous catalyst was used to produce linear alkanes from HDPE. This type of catalyst promotes a tandem reaction via which poorly reactive aliphatic substrates are first activated through dehydrogenation and then functionalized or cleaved by a highly active olefin catalyst [160].

These technologies are particularly attractive from an industrial point of view as heterogeneous catalysts are generally easier to use and economically more sustainable than homogeneous ones.

#### 4.2.3. Hydrosilylation

Hydrosilylation carried out in the presence of different silanes (tetramethyldisiloxane and polymethylhydrosiloxane) and borane or Ir catalysts has also been tested in the past for the depolymerization of PET, PS, and PVC [161]. Probably because of the high cost of reagents and Ir catalysts, combined with low yields in monomers recovered, no similar studies were published in the last 12 months. An interesting alternative was proposed by Fernandes and coworkers in 2020 for the depolymerization of PET by silanes and an air-stable, cost-effective dioxomolybdenum complex, MoO_2_Cl_2_(H_2_O)_2_. Although reaction conditions are rather harsh (160 °C, 4 days), very good yields in *p*-xylene were achieved for the reductive depolymerization of PET (65% yield) in the presence of 5 wt.% MoO_2_Cl_2_(H_2_O)_2_ and six equivalents of phenylsilane. In another study, Fernandes described the first example of reductive hydrosilylation of PET and other plastic waste using an economically and environmentally sustainable Zn catalyst, Zn(OAc)_2_·2H_2_O, to produce high-value-added compounds such as 1,2-propanediol, 1,6-hexanediol, tetrahydrofuran, and *p*-xylene. In the same reaction conditions, in the presence of Mo oxides, yields in *p*-xylene were equivalent while higher yields in EG were obtained (43%) [162]. Much work surely needs to be done to implement these technologies to industrial maturity, but the use of highly available, environmentally friendly catalysts is a great advantage and should be further pursued.

## 5. Thermal Recycling

**Scheme 6:**
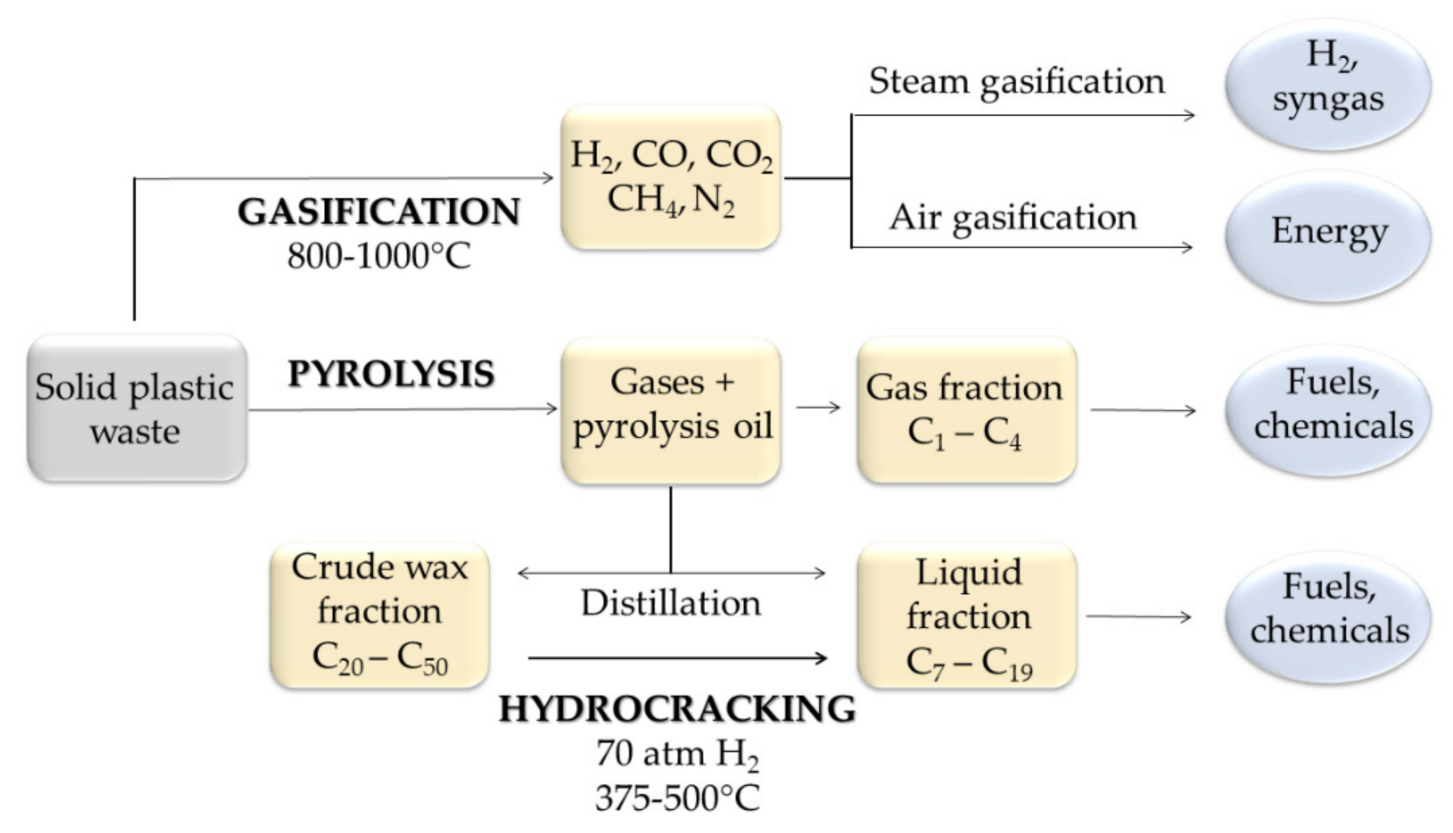
General scheme of thermal recycling processes.

Thermal recycling mainly comprises pyrolysis, hydrocracking, and gasification (Scheme 6) [163]. Since there are no recent advancements for gasification, only pyrolysis and hydrocracking are reported. An outline of the main innovative solutions recently published is reported below.

### 5.1. Pyrolysis

Pyrolysis, or thermal cracking, is a process that occurs at high temperatures (500 °C) and in the absence of oxygen. Different kinds of catalysts can be used to improve the efficiency of the pyrolysis process since they target a specific reaction and reduce the process temperature and time [164]. Unlike other thermochemical conversion methods, pyrolysis leads to liquid or wax mixtures rich in hydrocarbons, an ideal raw material for a refinery [165]. Thermal pyrolysis is typically used for the recycling of those polymers for which depolymerization is harsh and that are not currently mechanically recyclable (PE/PP/PS mixtures, multilayer packaging, and reinforced fibers). Thanks to the high temperatures, it guarantees molecular bond breaking in the polymer chains to give, depending on the nature of the polymer, depolymerization or random fragmentation [122,166]. Alternatively, catalytic pyrolysis can be performed on the same polymers at lower temperatures by carbocation formation and subsequent isomerization [161]. Both thermal and catalytic pyrolysis approaches are not selective, but advantages rely on high conversions, thermal stability of the products and, in some cases, high-value enriched oil production. Pyrolysis, therefore, is an interesting recycling approach for a safe circular economy [161,166].

Pyrolysis must be preceded by pretreatment of the plastic waste, to ensure that it is not contaminated by non-plastic materials such as metal and wood. This step is necessary to ensure the economic feasibility of the plastic-to-fuel (PTF) plant, and it can usually be achieved by sorting, crushing, or sieving depending on the origin of the waste. Since pretreatment techniques are consolidated methodologies, no innovative methods were reported in the last year.

Another important aspect derives from different sources of plastic processed which may be different in shape and size, requiring to be uniformly sized as grains before feeding into the pyrolysis process. This step adds an extra cost to the process.

Depending on the type of reactor, the pre-sizing step can be skipped or modified. For example, rotary kilns can accommodate differently sized and shaped plastics; hence, the pre-sizing step can be avoided. Fluidized bed reactors, instead, need to have uniform thermodynamics in the reactor; therefore, plastic waste should be evenly sized. To cope with this challenge, several feeding devices have been tested [166].

Currently, the study of catalytic pyrolysis is very active, and a wide range of synthetic catalysts have been employed to enhance the overall pyrolysis process and to improve the quality of produced liquid oil.

Most PE pyrolysis approaches are promoted by heterogeneous acid catalysts (e.g., zeolites, alumina, and silica) and are usually unselective, resulting in a broad distribution of gas (C3 and C4 hydrocarbons), liquid (cycloparaffins, oligomers, and aromatics), and solid products (char, coke). This behavior is due to the radical mechanism of the C–C bond scission, leading to a complex mixture of olefinic and cross-linked compound [122,166].

A very recent novel study on this topic was carried out by Miandad and coworkers, in which the effect on yield and product quality of Saudi natural zeolite was investigated [164]. Saudi natural zeolite catalyst was improved via novel thermal activation (TA-NZ) at 550 °C and acid activation (AA-NZ) with HNO_3_. Pyrolysis feedstock was composed of single or mixed PS, PE, PP, and PET, in the presence of both modified natural zeolite (NZ) catalysts. The authors reported that PS produced the highest yield in liquid oil, i.e., 70% and 60% using the TA-NZ and AA-NZ catalysts, respectively, compared to PP (40% and 54%) and PE (40% and 42%).

In addition to zeolite, the research on catalytic pyrolysis has focused on other catalytic systems, always considering that the catalytic activity of the catalyst is derived from its Lewis acid sites. Most homogeneous catalysts for polyolefin degradation have been classical Lewis acids such as AlCl_3_. On the basis of these considerations, Su and coworkers [167] worked on AlCl_3_–NaCl eutectic salt as a catalyst, allowing a reduction in reaction temperature, an increase in reaction rate, a reduction in heavy oil components, and the inhibition of polyolefin formation.

Pyrolysis is most often adopted to convert plastic waste to fuels. An example of differentiation is the production of high-value-added carbon nanotubes (CNTs) [168] using a metallic Ni catalyst supported on different oxides and generated in situ. Selectivity, yield, and structural properties were tuned according to the degree of metal–support interaction in different catalysts.

### 5.2. Hydrocracking

Hydrocracking is a catalytic refining process for the selective recovery of useful chemical fractions in the range of heavy diesel to light naphtha. Hydrocracking requires a bifunctional catalyst with an acidic function, enhancing the cracking activity, typically provided by a high-surface-area support, such as a zeolite [169].

Recent studies have focused on the conversion of both post-consumer and laboratory polymers in mild conditions, using a metal–zeolite catalytic system.

Jumah and coworkers [170] treated low- and high-density polyethylene (LDPE, HDPE), polypropylene (PP), and polystyrene (PS) to produce liquid petrol gas (C3–C4) and naphtha. They reported the effect of both the catalyst morphology (beta zeolite impregnated with 1% Pt) and the feed stream variation, by reacting different polymers individually and post-consumer polymer mixtures.

Another recent work described the transformation of PE, PP, and PS into methane (>97% purity) at 300–350 °C using near-stoichiometric amounts of H_2_ in the presence of a Ru-modified zeolite as a catalyst [171].

## 6. Conclusions

Ideally, the route to achieve a sustainable society is to replace synthetic plastics. A plastic-free world, however, is presently utopistic, and great effort must be applied in the pursuit of a drastic change in end-of-life plastic waste treatment and management.

In this review, we presented a highlight of the very latest technologies being developed to enhance the recycling efficiency of polymers and to generate high-value products from plastic waste.

Mechanical recycling and chemical upcycling appear to be the most promising strategies, since incineration and landfill are more pollutant and, for the latter, plastic waste completely loses its value.

Although, in the last few years, researchers have focused on chemical treatments, mechanical recycling is still the more mature and better performing technique. The lack of adequate infrastructures and technologies is limiting the industrialization of chemical upcycling, as well as the replacement of current materials with more sustainable polymers.

Future solutions will mainly focus on the development of biodegradable materials, completely recyclable polymers, and depolymerization/repolymerization pathways that allow to maximize the plastic life cycle.

Waste is a very serious problem and is intimately related to environmental and social–economic impacts. The problem of waste must be considered holistically from governments, industries, and stakeholders to preserve human health and guarantee the world survival. A deep change in mentalities at all levels is necessary to approach the impact of humanity and the industry on the environment; therefore, a high level of information is required to achieve awareness and promote sustainable processes and products. Too much information is available today; thus, that the scientific community must help give clear and well-justified indications regarding the best technologies to be adopted in the future. The authors hope that this mini-review will contribute to this consciousness and positively impact future choices.

## Figures and Tables

**Table 1:** Phosphine ligands of Milstein-type Ru–PNN complexes.

**Entry**	**Catalyst**	**Co-Cat.**	**Reaction Conditions**				**PET** **Conv. (%)**	**Sel. (%)**
			T (°C)	H_2_ (bar)	Solvent	t(h)		
**1**	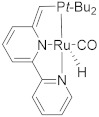	Kt-BuO	160	54	Anisole THF	48	99	100 ^1^
**2**	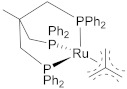	HNTf	140	100	Dioxane	16	42	64 ^1^
**3**	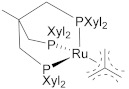	HNTf	140	100	Dioxane	16	64	99 ^1^
**4**	Ru/Nb_2_O_5_	/	220	/	H_2_O	12	91	100 ^2^
**5**	Ru/Nb_2_O_5_	/	200	3	H_2_O	12	95	100 ^2^

^1^ Selectivity to BDM. ^2^ Selectivity to BTX.

## Data Availability

Not applicable.
